# Fennel for Reducing Pain in Primary Dysmenorrhea: A Systematic Review and Meta-Analysis of Randomized Controlled Trials

**DOI:** 10.3390/nu12113438

**Published:** 2020-11-10

**Authors:** Hye Won Lee, Lin Ang, Myeong Soo Lee, Zainab Alimoradi, Eunseop Kim

**Affiliations:** 1 Herbal Medicine Research Division, Korea Institute of Oriental Medicine, Daejeon 34054, Korea; hwlee@kiom.re.kr; 2 Clinical Medicine Division, Korea Institute of Oriental Medicine, Daejeon 34054, Korea; anglin2808@kiom.re.kr; 3 Korean Convergence Medicine, University of Science and Technology, Daejeon 34113, Korea; 4 Social Determinants of Health Research Center, Research Institute for Prevention of Non-Communicable Diseases, Qazvin University of Medical Sciences, Qazvin 3419759811, Iran; zainabalimoradi@yahoo.com; 5 You and Green Korean Medicine Clinic, Daejeon 35262, Korea; greenmiz@naver.com

**Keywords:** fennel, extract, placebo, pain, dysmenorrhea, systematic review, meta-analysis

## Abstract

Fennel is used as an alternative treatment for primary dysmenorrhea. This review aims to evaluate the effectiveness and safety of fennel for reducing pain in primary dysmenorrhea. Twenty databases, including English, Korean, Chinese, Japanese, Iranian, and Spanish databases, were searched from inception to 20 October 2020. All randomized controlled trials (RCTs) investigating the effectiveness of fennel for treating primary dysmenorrhea were considered. Two reviewers conducted the data extraction and risk of bias assessment independently. Any discrepancies were resolved through discussion with a third reviewer. A total of 12 studies were included in this review. The pooled results of seven trials showed that the effect of fennel is similar to that of conventional drug therapies in alleviating pain (*n* = 502, standardized mean difference (SMD): 0.07, 95% confidence interval(CI): −0.08 to 0.21, *p* < 0.37, *I*^2^ = 0%). In comparison with placebo, fennel was seen to have favorable effects on reducing pain in primary dysmenorrhea (*n* = 468, SMD: −3.27, 95% CI: −5.28 to −1.26, *p* = 0.001, *I*^2^ = 98%). Only three studies assessed adverse events (AEs), and one study reported minor AEs. Although the risk of bias for all the included studies was moderate, potential publication bias was observed due to the presence of a greater number of small studies with favorable effects. This systematic review concludes that fennel is as effective as conventional drug therapies in alleviating pain in primary dysmenorrhea. More studies that include more diverse populations and robust evidence of fennel’s effects will be needed in future research endeavors.

## 1. Introduction

Primary dysmenorrhea, commonly known as menstrual cramps, is a recurrent pain around the lower abdomen occurring immediately before or during menstruation in the absence of underlying conditions, and it is often accompanied by symptoms such as fatigue, headaches, dizziness, nausea, sweating, and diarrhea [1]. It is a common gynecological condition, and approximately 45 to 95% of menstruating women suffer from it [2]. Dysmenorrhea negatively affects the quality of life of those affected, including school/work performance or productivity, relationships between sufferers and their family or friends, as well as participation in recreational or social activities [3,4,5,6,7]. Reduced productivity, medication costs, and the need for medical treatment due to dysmenorrhea also lead to substantial economic losses [8].

Nonsteroidal anti-inflammatory drugs (NSAIDs) such as mefenamic acid are the first-line pharmacological treatments for dysmenorrhea [1]. Although the conventional treatments (NSAIDs and oral contraceptives (OCPs)) are effective in relieving menstrual pain, there has been an increase in the risk of adverse effects, such as mild gastrointestinal and neurological symptoms [9,10]. In addition to pharmacological agents, many consumers and practitioners use complementary and alternative medicine to treat painful menstruation [11]. Herbal therapies, one of the complementary medicines widely used for dysmenorrhea, are considered supplements in the United States [12].

Fennel or *Foeniculum vulgare* is an herbal therapy that is proposed to alleviate menstrual pain by lowering the prostaglandin levels in blood [13]. A few studies have used fennel as an alternative therapy for the treatment of primary dysmenorrhea. This review aims to assess the effectiveness and safety of fennel for reducing pain in primary dysmenorrhea.

## 2. Methods

This systematic review of the literature concerning fennel’s effects on primary dysmenorrhea was conducted according to Preferred Reporting Items for Systematic Reviews and Meta-Analyses (PRISMA) guidelines and was registered with the Research Registry (unique identifying number: reviewregistry1025) [14].

### 2.1. Data Sources

We performed literature searches in 20 databases from their inception to 20 October 2020: 4 English databases (MEDLINE, AMED, EMBASE, Cochrane Library), 4 Spanish databases (BibliMed, IBECS, Medes, and Guia Salud), Virtual Health Library (VHL, Biblioteca Virtual en Salud, by Latin American and Caribbean Center on Health Sciences Information), 4 Iranian databases (SID, IranDoc, Magiran, and IranMedex), 3 Korean medical databases (Korean Studies Information, Research Information Service System, and KoreaMed), 3 Chinese databases (CNKI, VIP, and WanFang), and 1 Japanese database (J-Stage).

We used the following search terms: (fennel OR *Foeniculum vulgare*) AND (dysmenorrhea OR primary dysmenorrhea OR menstruation disturbances OR painful menstruation OR period pain OR painful period OR pelvic pain OR menstrual disorder) AND (randomized controlled trial) in English, Persian, Spanish, Chinese, and Korean. The detailed search strategy for MEDLINE is provided as supplementary material (Supplementary Materials). In addition, we hand-searched the reference lists of all the retrieved articles for further relevant literature. The authors of the included studies, and experts in the field when necessary, were contacted about unpublished data. We read hard copies of all the included articles in full.

### 2.2. Study Selection

#### 2.2.1. Types of Studies

We included all randomized controlled trials (RCTs) regardless of trial designs, such as quasi-RCTs, cluster-RCTs, pseudo-RCTs, and randomized crossover trials, and we excluded observational studies, cohort studies, case–control studies, case series, qualitative studies, uncontrolled trials, and laboratory studies.

#### 2.2.2. Types of Participants

We included trials that investigated women of reproductive age (aged 15 to 49 years) with primary dysmenorrhea, i.e., absence of pelvic pathology identified by pelvic examination, ultrasound scans, or laparoscopy, as well as self-reported primary dysmenorrhea. The exclusion criteria were identifiable pelvic pathology and dysmenorrhea due to the usage of an intrauterine contraceptive device.

#### 2.2.3. Types of Interventions

We included trials that involved the oral use of fennel for managing dysmenorrhea, including capsule, oil, or pill. We excluded massage with fennel oil. There was no limitation on the dosage, the forms of fennel, or the duration of treatment.

#### 2.2.4. Types of Comparisons

We included trials that used drug therapies or placebo as a control intervention. We excluded trials with waiting lists, no treatment, and complementary and alternative-related therapies.

#### 2.2.5. Outcome Measures

Primary outcome

− The primary outcome was pain, especially the reduction in menstrual pain occurring during trial intervention or as an intervention result, which was measured using validated scales such as the visual analog scale (VAS). The pain outcome presented in the form of dichotomous outcomes was also included.

Secondary outcome

− Adverse events (AEs) were studied as secondary outcome.

### 2.3. Data Extraction and Risk-of-Bias Assessment

Two independent reviewers read all articles and extracted the data along with the predefined criteria. We obtained information regarding the participants, interventions, outcome measures, and results from each study. Any disagreements between the two authors were resolved by discussion with another author (MSL) acting as an arbiter. The Iranian papers published in Persian were translated into English by the co-author. The risk of bias (ROB) was assessed using the following six domains from the Cochrane risk of bias tool (version 1.0) [15]: (1) generation of random sequence, (2) concealment of allocation, (3) blinding of participants and personnel, (4) blinding of outcome assessment, (5) incomplete outcome data, and (6) selective outcome reporting. This review used “Low” indicating a low ROB (L), “Unclear” indicating an uncertain ROB (U), and “High” indicating a high ROB (H) as judgment keys. Disagreements were resolved by discussion among all the reviewers.

### 2.4. Data Synthesis

We used the Cochrane Collaboration’s software program, Review Manager (RevMan), version 5.3.0 for Windows (Copenhagen, The Nordic Cochrane Center, Copenhagen, Denmark) to conduct all statistical analyses. Differences between both the intervention and control groups were calculated. To analyze the standardized mean difference (SMD) with 95% confidence intervals (CIs) between both groups, the generic inverse variance method in RevMan was applied. SMD was applied as all the included studies measured the same outcome using different measuring scales, e.g., assessment of pain using different scales across the studies. We also contacted the primary authors, where possible, to obtain and validate information from studies lacking data. We then pooled the data across the studies using random-effects models when appropriate. The chi-square (χ^2^) test for heterogeneity and the *I*^2^ test were used to assess the heterogeneity of the included studies. We also intended to conduct subgroup analyses according to the different forms of fennel. A funnel plot was used to investigate publication bias. We generated an albatross plot with *p*-value, sample size, and direction of results to complement the results from the meta-analysis of available effects using Stata (StataCorp LLC, College Station, TX, USA) and Albatross module [16,17].

## 3. Results

### 3.1. Description of the Included Trials

**Figure 1:**
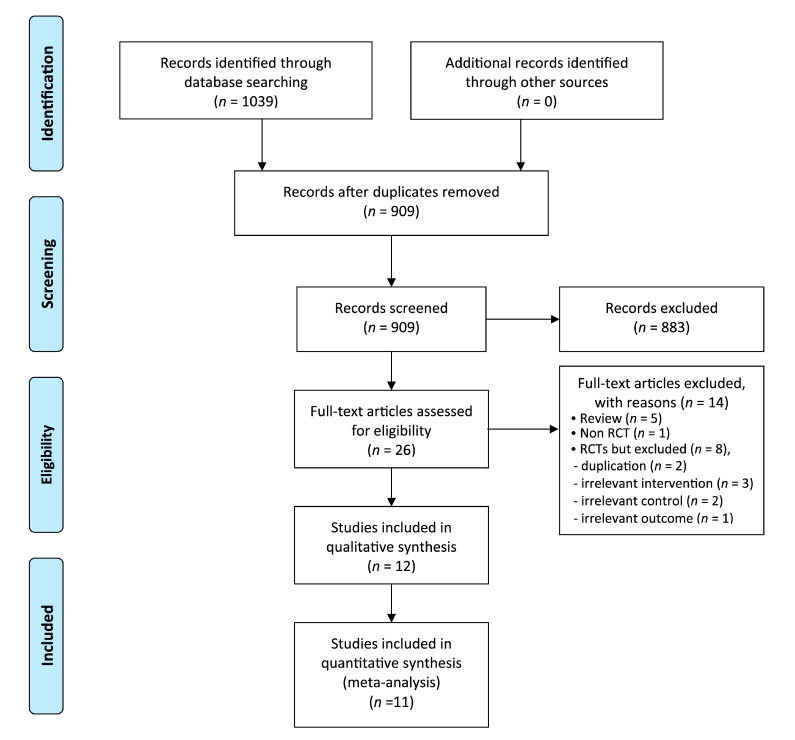
Flow diagram of the selection process. RCT: randomized controlled trial.

We identified 1039 potentially relevant records, 12 of which met our inclusion criteria (Figure 1). The studies’ characteristics are summarized in Table 1 [18,19,20,21,22,23,24,25,26,27,28,29]. Nine RCTs tested the oral use of fennel oil [18,19,20,21,22,23,24,28,29], and the other three studies tested the use of the capsule type of fennel [25,26,27]. All the included trials originated from Iran. Eleven studies used a parallel design [18,19,20,21,22,23,24,25,26,27,29], and one study used a crossover design [28]. Seven studies compared fennel to drug therapy [18,19,20,21,22,23,24], and seven RCTs included placebo as a control [23,24,25,26,27,28,29].

### 3.2. Risk of Bias (ROB)

**Figure 2:**
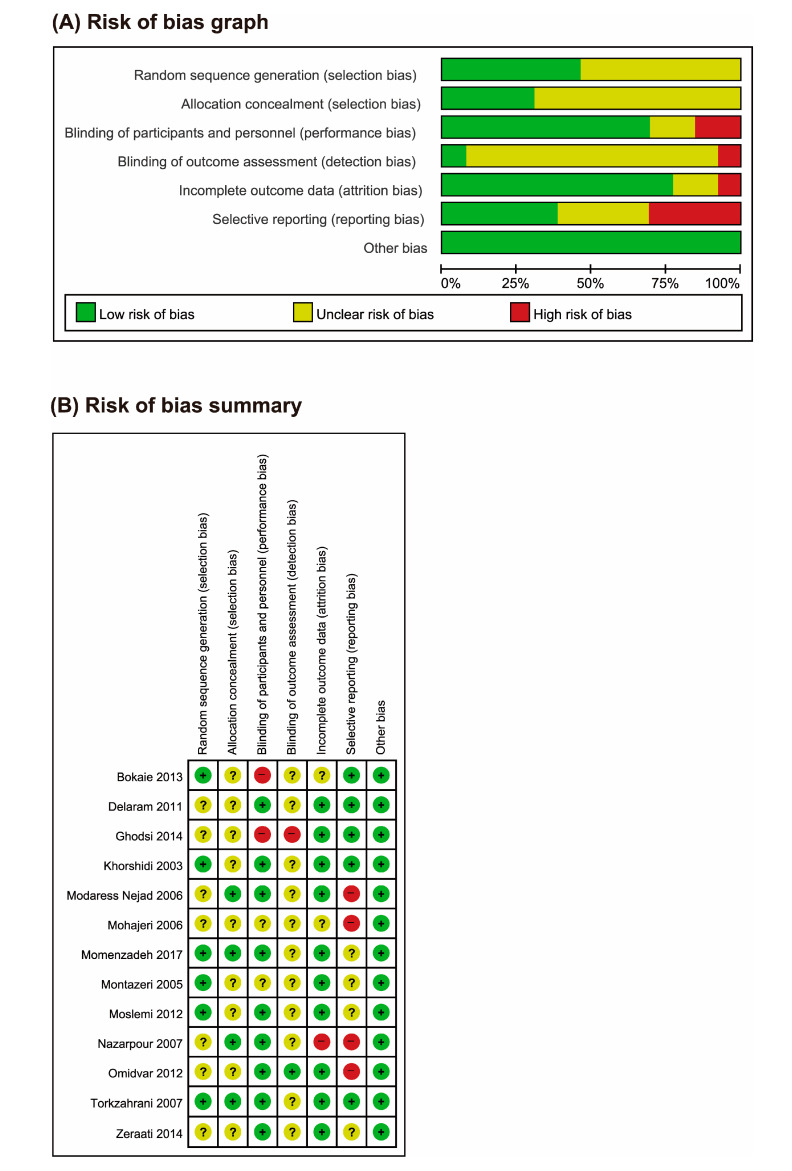
(**A**) Risk of bias graph: review authors’ judgments about each risk of bias item presented as percentages across all included studies. (**B**) Risk of bias summary: review authors’ judgments about each risk of bias item for each included study. +: low risk of bias; −: high risk of bias; ?: unclear risk of bias.

The ROB was generally moderate in the included trials (Figure 2). Six studies used adequate sequence generation [18,21,22,25,27,28], and only four described allocation concealment [19,21,23,27]. Nine studies used controls which had identical shape to fennel treatment [19,21,23,24,25,26,27,28,29]. Four RCTs failed to report their results completely [19,20,23,26].

### 3.3. Outcome Measurements

#### 3.3.1. Pain

##### Fennel vs. Drug Therapies

**Figure 3:**
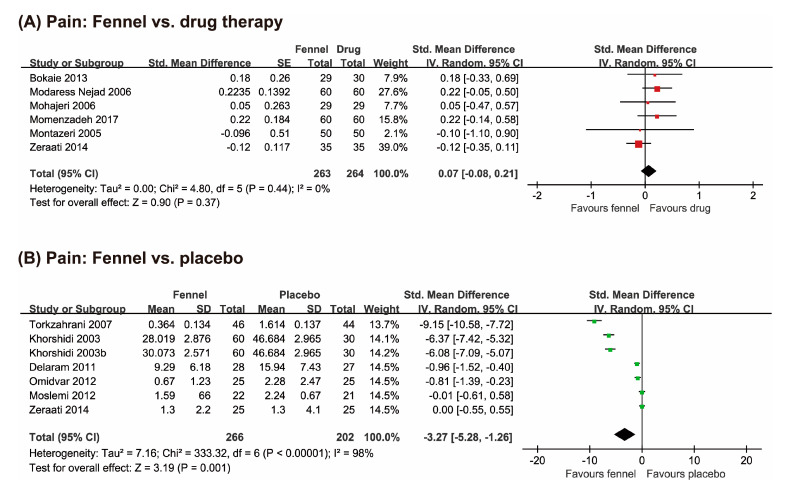
Forest plot of effects of fennel on menstrual pain compared with (**A**) drug therapies and (**B**) placebo. CI: confidence interval; SD: standard mean difference.

Seven RCTs investigated the effects of fennel for treating dysmenorrhea compared to drug therapy [18,19,20,21,22,23,24]. All trials reported equivalent effects of fennel on pain reduction compared with conventional drug therapy. The meta-analysis also showed the equivalent effects of fennel on pain reduction compared with drug therapy (*n* = 502, SMD: 0.07, 95% CI: −0.08 to 0.21, *p* < 0.37, *I*^2^ = 0%, Figure 3A).

##### Fennel vs. Placebo

Seven studies compared the efficacy of fennel for pain reduction to placebo [23,24,25,26,27,28,29]. Five RCTs showed superior effects of fennel [23,26,27,28,29], while two studies failed to do so [24,25]. The pooled results showed favorable effects of fennel on pain reduction compared to placebo (*n* = 468, SMD: −3.27, 95% CI: −5.28 to −1.26, *p* = 0.001, *I*^2^ = 98%, Figure 3B).

#### 3.3.2. Adverse Events (AEs)

Only three trials assessed AEs [21,24,28], whereas the other nine did not. One study reported that no AEs were observed [21]. One study failed to report the AEs in detail [28], while the other study reported nausea and vomiting in the fennel and placebo groups [29].

#### 3.3.3. Publication Bias and Albatross Plot

**Figure 4:**
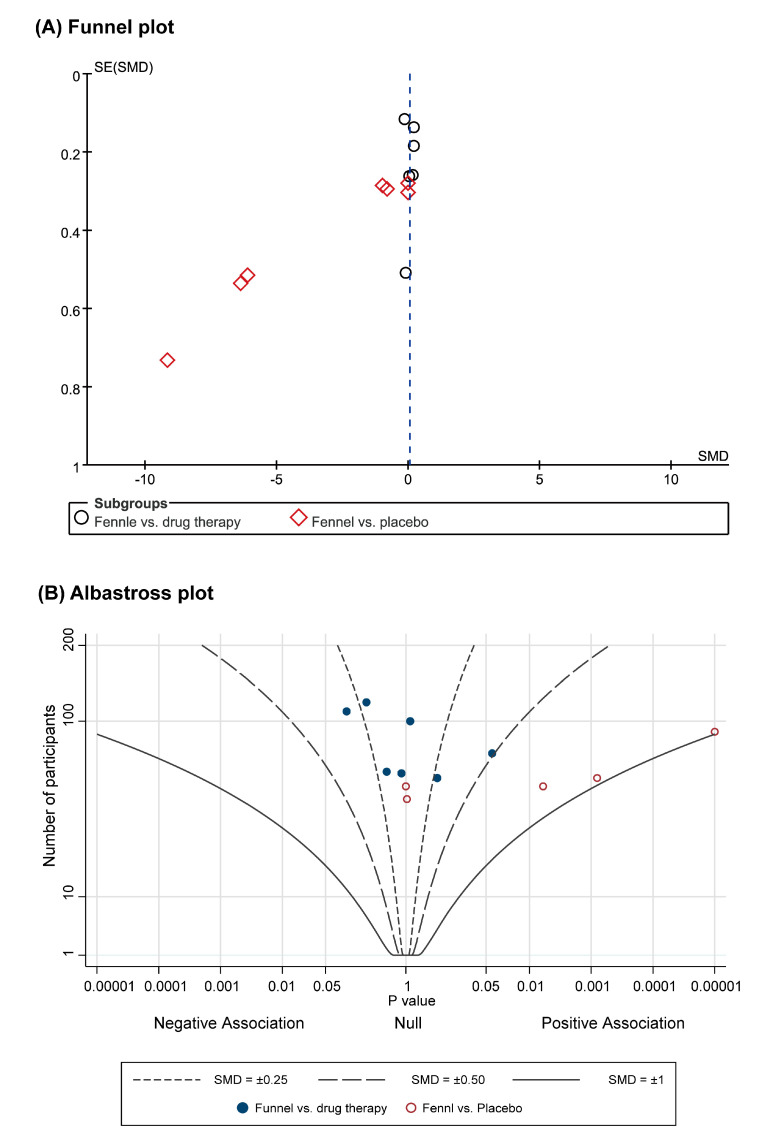
(**A**) Funnel plot for fennel for managing pain; (**B**) Albatross plot. SE: standard err; SMD: standard mean difference.

Funnel plots were asymmetrical for the SMD of pain, presenting potential publication bias (Figure 4A) due to the presence of more studies with smaller sample sizes presenting favorable effects.

The albatross plot showed positive associations of funnel on dysmenorrhea compared to placebo control, whereas the data for drug therapies showed null association (Figure 4B).

## 4. Discussion

### 4.1. Summary of the Main Results

We identified 12 RCTs that addressed the effects of fennel on reducing pain in primary dysmenorrhea. The meta-analysis indicated that fennel was as effective as conventional drug therapies in reducing pain in primary dysmenorrhea, compared to placebo. However, fennel was found to be significantly superior to placebo in the reduction of pain. As the majority of the studies did not assess AEs, we cannot provide judgment on the safety of fennel in treating primary dysmenorrhea.

### 4.2. Overall Completeness and Applicability of the Evidence

This review has several limitations regarding overall completeness and applicability. These limitations arose from the criteria of the included studies. The overall severity of dysmenorrhea of the study participants varied between studies, from mild to severe. However, available data did not allow an evaluation of whether the effectiveness of fennel differs with the severity of illness. In addition, the treatment duration of the included studies ranged from one-time treatment to six months, and the monitoring of AEs was lacking in the majority of the studies. The findings from these observation periods did not allow an exploration of the optimal treatment duration for clinical application. Additionally, the number of studies included was small. The effect estimates per comparison did not allow subgroup analysis to compare the differential treatment effects of the different forms and dosages of fennel. Publication bias was also observed due to the presence of a greater number of small studies that showed favorable effect. This resulted in the asymmetrical shape of the funnel plot.

### 4.3. Certainty of the Evidence

The overall robustness of the findings is considered low. The comparability and interpretability of the results were challenging, and high statistical heterogeneity was observed in part of our findings, as different assessment instruments were used for the same outcome variable. In addition, the certainty of the results of this review may be downgraded by the relatively small numbers of included studies and study participants. The sample size of each included study was rather small. Although we pooled the effect estimates of the studies, the power to detect significance remained relatively low. Hence, further trial findings are likely to affect the results of this review. In our ROB assessment, only a few studies were judged to have adequate allocation concealment, blinding, and outcome reporting, even though the overall ROB was moderate. Despite our attempts to avoid publication bias by searching for all relevant studies, potential publication bias was detected in the funnel plot.

### 4.4. Potential Biases in the Review Process

Many studies on fennel have been published in Middle Eastern countries in recent years, particularly in Iran. There are certain limitations in assessing Middle Eastern countries’ databases, such as identifying the appropriate core database, requiring the literature search to be performed in the Persian language, and the lack of English titles and abstracts for the screening of study eligibility. These factors may impede the retrieval of all trials. Although we tried to perform a thorough and comprehensive search, there is the possibility that eligible trials were overlooked. Furthermore, some articles are translated articles where there might be a misinterpretation of the language, even though professional translation services were used. Although such misinterpretation will not affect the outcome data of the included studies, it might have influenced the quality assessment and the full understanding of the studies. In addition, the majority of included trials lacked trial registration, which reduced the reliability of the included studies. All of the studies included in this review were also conducted in Iran, and the funnel plot showed possible publication bias. Hence, the generalization of the results to other countries might be limited.

### 4.5. Agreements and Disagreements with Other Studies or Reviews

Several systematic reviews of interventions related to fennel for dysmenorrhea have been published [30,31,32,33,34,35]. Three of them focused on fennel for primary dysmenorrhea [30,31,33], while the other four analyzed several interventions in one review [32,34,35,36,37]. One recent review evaluated the efficacy of fennel based on nine studies and concluded that fennel extract had a positive effect on menstrual pain [31]. However, this review included one non-RCT and one duplicated publication. Additionally, this review pooled the data regardless of controls. This may exaggerate the efficacy and lead to misinformation by ignoring the clinical heterogeneity. The second systematic review analyzed five studies and claimed that further studies on fennel’s effects were necessary [33]. However, our review included more than double the number of studies included in the second review, thereby providing more robust evidence. The third review included 13 studies and concluded that the effects of fennel on primary dysmenorrhea were positive [30]. However, this review did not consider the study design or type of controls. Furthermore, the second and third reviews did not assess the ROB and did not apply it in the interpretation of the evidence [30,33]. Compared with these previous systematic reviews, we identified a total of three new RCTs [20,21,22] and comprehensively updated the evidence for fennel with rigorous methods. We analyzed the efficacy of fennel by comparing both the drug and placebo. Our review showed that fennel is effective for reducing menstrual pain compared with placebo and has equivalent efficacy to conventional drug therapies.

### 4.6. Implication for Clinical Practice

To date, the research base lacks firm evidence that fennel provides a benefit to people suffering from primary dysmenorrhea, as well as the potential extent of this benefit, and more robust findings are certainly desirable. Nevertheless, our results show that fennel is as effective as conventional drug therapies and can be recommended as an effective treatment. This conclusion, derived from the studies included in this review, is drawn with caution, considering the limitations discussed above.

### 4.7. Implications for Research

To enhance the robustness of the findings, rigorous studies are highly warranted. All clinical trials should be registered, as study registration can substantially improve transparency in clinical trials, prevent reporting bias, and reduce selective reporting. In addition to efficacy, outcome assessments should include a standardized assessment of AEs for future safety evaluation. Researchers and authors should also refer to reporting guidelines to ensure that their studies are reported appropriately and to increase the replicability of their studies.

## 5. Conclusions

This systematic review presents evidence on the potential effectiveness of fennel in treating primary dysmenorrhea. Our meta-analysis indicates that fennel is as effective as conventional drug therapies in reducing pain in primary dysmenorrhea, compared to placebo. Although the efficacy of fennel was reported by all twelve studies, only two studies reported on its safety. Future studies should assess and report on its safety, in addition to its efficacy.

## Figures and Tables

**Table 1:** Summary of randomized clinical trials of fennel for dysmenorrhea.

First Author (Year) [Ref]	Sample Size/Analyzed Age (Year) Severity	Intervention	Control	Treatment Duration	Main Outcome	Result	Design AEs Registration No.
Bokaie (2013) [18]	60/59 8–25 years moderate to severe	(A) Fennel (oil, oral, 2%, 25 drops/6 h, from beginning of pain or 1st menstrual day, *n* = 29), plus (B)	(B) Mefenamic acid (250 mg, *n* = 30)	1 month	Pain (VAS)	MD 0.40 [−0.74, 1.54], NS	Parallel Not assessed IRCT 201107096826N2
Modaressnejad (2006) [19]	120/110 13–18 n.r.	(A) Fennel (oil, oral, 30 drops/6 h, *n* = 55)	(B) Mefenamic acid (250 mg/6 h, *n* = 55)	2 consecutive months (first three days of menstruation)	Pain (AMVMS)	RR 1.10 [0.89, 1.36], NS	Parallel Not assessed NA
Mohajeri (2006) [20]	58/58 18–28 n.r.	(A) Fennel (oil, oral, 25 drops, 3 times day, *n* = 29)	(B) Mefenamic acid (500 mg loading dose then 250 mg, 3 times daily, *n* = 29)	6 consecutive months	Pain (VAS)	RR 0.96 [0.79, 1.16], NS	Parallel Not assessed NA
Momenzadeh (2017) [21]	120/120 18–23 moderate to severe	(A) Fennel (oil, oral, capsule, 30 mg, *n* = 60)	(B) Mefenamic acid (250 mg capsule, *n* = 60)	2 consecutive months (3 days before onset of menstruation up to first three days)	Pain (AMVMS)	MD 0.13 [−0.08, 0.34], NS	Parallel No AEs reported NA
Montazeri (2005) [22]	120/100 15–19 moderate to severe pain	(A) Fennel (oil, oral, 2%, 20–30 drop/4–6 h, *n* = 50)	(B) Ibuprofen (400 mg, per 4–6 h, *n* = 50)	3 consecutive months (first month with no intervention followed by 2 months of intervention)	Pain (AMVMS)	RR 0.94 [0.71, 1.25], NS	Parallel Not assessed NA
Nazarpour (2007) [23]	120/104 17–25 moderate to severe	(A) Fennel (oil, oral, 2%, 20–30 drops/4 to 8 h, *n* = 36)	(B) Mefenamic acid (250 mg/6 h, *n* = 36) (C) Placebo (same shape and same order, *n* = 32)	2 consecutive months	Pain (VAS)	A vs. B: MD −0.58 [NA], *p* < 0.05 A vs. C: MD −0.09 [NA], NS	Parallel Not assessed NA
Zeraati (2014) [24]	105/105 18–25 mild to moderate	(A) Fennel (oil, oral, 30 drops/4 h, *n* = 25)	(B) Mefenamic acid (capsules 250 mg/4 h, *n* = 30) (C) Placebo(30 drops/4 h, *n* = 25) * (D) Vitagnus (40 drops daily in the morning, *n* = 25)	3 consecutive months (1 day before the start of the cycle until the third day for three cycles: one without any drugs and then two cycles with them)	Pain (VAS)	A. vs. B: MD −0.38 [−1.93, 1.17], NS A. vs. C: MD 0.00 [−1.82, 1.82], NS	Parallel Not assessed NA
Moslemi (2012) [25]	65/63 n.r.Moderate to severe	(A) Fennel (extract, capsule, 46 mg, *n* = 22)	(B) Placebo (*n* = 21) * (C) Vt E (*n* = 20)	2 consecutive months (every 6 h for 3 days for two consecutive cycles)	Pain (AMVMS)	MD −0.65 [−1.02, −0.28], *p* < 0.001	Parallel Not assessed IRCT201106046705N1
Omidvar (2012) [26]	50/50 15–24 Moderate to severe	(A) Fennel (extract, capsules, 30 mg, *n* = 25)	(B) Placebo (capsules, wheat flour, *n* = 25)	2 consecutive months (four times daily for 3 days for two consecutive cycles)	Pain (VAS)	MD −1.61 [−2.69, −0.53], *p* < 0.01	Parallel Not assessed NA
Torkzahrani (2007) [27]	130/90 17–30 moderate to severe	(A) Fennel (46 mg/capsule, 5 capsules daily, *n* = 46)	(B) Placebo (same shape and same order, *n* = 44)	2 consecutive months (from the 1st day of menstrual bleeding, capsules were administered only for first three days)	Pain (AMVMS)	MD −1.25 [−1.31,−1.19], *p* < 0.001	Parallel Not assessed NA
Khorshidi (2003) [28]	60/55 17–25 mild to moderate	(A) Fennel (oil, oral, 1%, *n* = 18) (B) Fennel (oil, oral 2%, *n* = 19)	(C) Placebo (n.r., *n* = 16)	1 time, administrated as soon as pain felt	Pain (Likert scale)	A vs. C: MD −18.66 [−20.48, −16.85], *p* < 0.001 B vs. C: MD −16.61 [−18.42, −14.80], *p* < 0.001	Cross-over AE reported (no details) NA
Delaram (2011) [29]	60/55 8–25 Severe	(A) Fennel (oil, oral, 30 drops/8 h, *n* = 28)	(B) Placebo (same shape and same order, *n* = 27)	2 consecutive months (3 days before onset of menstrual bleeding to three first days of bleeding)	Pain (VAS)	MD −6.65 [−10.27, −3.03], *p* < 0.001	Parallel AE reported Nausea and vomiting (A:1; B:1) NA

AMVMS: Andersch and Milsom’s verbal multi-dimensional scoring system; AE: adverse events; IRCT: Iranian Registry of Clinical Trials; MD: mean difference; NA: not available; n.r.: not reported; NS: not significant; RR: risk ratio; VAS: visual analog scale; VRS: verbal rating scale. * This group was not considered for analysis because of not falling into review inclusion criteria. [ ]: inside values are indicated confidence intervals.

## Data Availability

Information used for this review is included in the article and provided as references. Any other data will be made available upon request.

## Supplementary Materials

The following are available online at https://www.mdpi.com/2072-6643/12/11/3438/s1.
