# Supplementary material for: Fennel for Reducing Pain in Primary Dysmenorrhea: A Systematic Review and Meta-Analysis of Randomized Controlled Trials
Source: Nutrients. 2020 Nov 10;12(11):3438. doi: 10.3390/nu12113438 (PMC7697926; doi:10.3390/nu12113438)
Supplement: Supplementary file 1 [file nutrients-12-03438-s001.pdf]

Supplement 1. Search Strategy for MEDLINE® <1946 to present>

1 exp dysmenorrhea/

2 pain$ period$.tw.

3 menstrua$ cramp$.tw.

4. menstrua$ disturb$.tw.

5. menstrua$ disorder$.tw.

6. pelvi$ pain$.tw.

7. (period$ adj3 cramp$).tw.

8. (menstrua$ adj3 pain$).tw.

9. dysmenorrh$.tw.

10. or/1-9

11. exp fennel/

12. exp Foeniculum vulgare/

13. or/11-12

14. randomized controlled trial.pt.

15. controlled clinical trial.pt.

16. randomized.ab.

17. randomised.ab.

18. clinical trials as topic.sh.

19. randomly.ab.

20. trial.ti. (124986)

21. or/14-20

22. 10 and 13 and 21
